# Regional and residential disparities in knowledge of abortion legality and availability of facility-based abortion services in Ethiopia

**DOI:** 10.1016/j.conx.2021.100066

**Published:** 2021-06-22

**Authors:** Grace Sheehy, Jessica L. Dozier, Alexandria K. Mickler, Mahari Yihdego, Celia Karp, Linnea A. Zimmerman

**Affiliations:** a Department of Population Family and Reproductive Health, Johns Hopkins Bloomberg School of Public Health, Baltimore, MD, United States; b PMA-Ethiopia, Addis Ababa University, Addis Ababa, Addis Ababa, Ethiopia

**Keywords:** Abortion, Abortion laws, Ethiopia, Legal knowledge

## Abstract

**Objectives:**

To generate regional, residential, and nationally representative estimates of knowledge of abortion legality and availability among women of reproductive age in Ethiopia, and examine how knowledge varies across regions and by urban/rural residence.

**Study Design:**

Our study draws on data from a nationally representative, cross-sectional survey implemented in 2019 in each of Ethiopia's regional and administrative states, yielding a sample of 8,837 women aged 15 to 49. We compare weighted estimates and regional distributions of 3 outcomes: (1) general awareness and (2) correct knowledge of the abortion law, and (3) knowledge of facility-based abortion service availability.

**Results:**

Significant regional and urban/rural disparities in knowledge of abortion legality and availability exist. Nationally, 27% of women are aware of the abortion law and just 5% of women have comprehensive knowledge of the law, while 30% know where to access facility-based abortion services. Regionally, estimates range significantly, from 2% in Somali to 45% in Addis Ababa for general awareness of the law, 0% in Afar to 27% in Harare for comprehensive knowledge of the law, and 8% in Afar to 57% in Tigray for knowledge of abortion availability. Knowledge of all measures is higher in urban than in rural areas.

**Conclusions:**

Regional disparities in abortion knowledge may contribute to geographic inequities in access to and use of safe abortion care. Efforts to expand knowledge of abortion legality and availability are needed and should be tailored to regional contexts.

**Implications:**

Knowledge of abortion legality and availability is imperative to protecting and expanding access to safe abortion care, especially in contexts like Ethiopia where abortion is available for multiple indications. Efforts to improve knowledge of abortion legality and availability are needed, and should be locally tailored to address regional inequities.

## 1 Introduction

Globally, an estimated 22 million unsafe abortions occur annually, with approximately 97% in developing countries [1]. Access to safe abortion depends on multiple factors, including the legal and political context of abortion, stigma, access to information, and the capacity of health systems to provide high-quality reproductive health services [1]. Importantly, utilization of safe abortion services varies by women's awareness and knowledge of abortion legality [2], [3], [4]. Ensuring women can make informed decisions regarding their reproductive health is an essential component of promoting universal access to sexual and reproductive health and rights, an indicator within Target 5.6 of the Sustainable Development Goals [5]. Thus, knowledge of the right to safe, legal abortion care is important for ensuring services are available to the fullest extent of the law [6].

An estimated 620,300 abortions were performed in Ethiopia in 2014 [7]. In 2005, Ethiopia amended its abortion law, legalizing abortion among minors and in cases of rape, incest, fetal impairment, or maternal disability [8]. Surgical and medication abortion services are offered by a range of providers in health facilities, and use of medication abortion has increased considerably in recent years, rising from 0% to 36% of all abortion procedures between 2008 and 2014 [9, 10]. Despite government efforts to improve access to facility-based abortion care [8, 11], regional disparities persist. Comprehensive abortion care is disproportionately offered in smaller administrative regions (including Harare, Dire Dawa, Gambella, Addis Ababa, and Tigray), resulting in limited access to safe abortion for women living in larger regions [12]. Five regions do not contain the recommended number of facilities for provision of safe abortion care relative to population size, and over 70% of facilities in Afar, Gambella, and Oromiya do not perform first trimester abortions [12].

Nearly half of abortions in Ethiopia occur outside of health facilities, often under unsafe conditions [7]. Although the proportion of women accessing facility-based abortion (including both medication and surgical abortion) increased from 27% to 53% since 2008, rates of abortion-related complications remain high, suggesting unsafe abortion is still common [7, 10], and several studies of highly selective populations indicate limited knowledge of the legal grounds for abortion among Ethiopian women [2, [13], [14], [15], [16], [17], [18]]. In settings like Ethiopia where abortion is available for multiple indications, ambiguity often exists about the circumstances under which abortion is legal. Evidence from other countries, including Ghana and Nepal, suggests that lack of knowledge of abortion legality can impede women's access to safe, timely abortion care [3, 4]. To date, no nationally or regionally representative estimates of women's knowledge of abortion legality in Ethiopia exist. Given Ethiopia's regional diversity and disparities in abortion availability, research exploring regional variation in knowledge of abortion legality and availability is necessary to inform tailored approaches to improve safe abortion care across the country.

We aim to generate representative estimates of national and regional knowledge of abortion legality and service availability among women of reproductive age in Ethiopia and examine regional and urban and/or rural variation in knowledge of abortion legality and availability.

## 2 Methods

### 2.1 Study design

Our study draws on data collected by Performance Monitoring for Action (PMA) Ethiopia. PMA Ethiopia is a 5-year project implemented collaboratively by Addis Ababa University (AAU) and Johns Hopkins University (JHU). PMA Ethiopia conducts nationally representative surveys measuring a range of key reproductive, maternal, and newborn health indicators. The study design and survey administration have been described in detail elsewhere [19].

Data come from a cross-sectional survey of women aged 15 to 49, collected from October through December 2019. A 2-stage cluster sampling design was used, and data were collected in each of 9 regional states and 2 administrative states. A total of 265 geographic enumeration areas (EAs) were selected by the Central Statistical Agency, and a cross-section of 35 households were selected randomly within each EA. Women were eligible to complete the survey if they were aged 15 to 49 years, lived within the selected EA boundaries, and slept in the selected household the night prior to the survey. Participants provided oral informed consent. Altogether, 8890 women from 9202 households participated in the female survey, with a response rate of 98.4%; our final analytic sample included 8837 women from whom we had complete data on the outcomes of interest (knowledge of abortion legality and availability), as well as current contraceptive use. The study received ethics approval from the JHU and AAU Institutional Review Boards.

### 2.2 Measures

Our 3 dependent variables comprised (1) general awareness of abortion legality, (2) comprehensive knowledge of the national abortion law, and (3) knowledge of facility-based abortion service availability.

We assessed awareness and knowledge of the abortion law using responses to 2 survey questions. First, we asked participants if they knew about a law regulating abortion in Ethiopia. Per the definition used by Assifi et al. in their systematic review on women's knowledge of abortion laws, we consider this question to measure women's *general awareness* of abortion legality [2]. Women who replied affirmatively were then asked to indicate under which circumstances it is legal to have an abortion in Ethiopia. The response options were read aloud to participants and included: (1) rape, when the (2) pregnancy poses a risk to the life of the woman and/or fetus (risk to the woman), (3) fetus has been diagnosed with an incurable disease or serious deformity (fetal impairment), (4) pregnant woman is incapacitated, or physically/mentally unfit to be a mother (maternal disability), and (5) under no circumstances. We used women's responses to these items to measure *comprehensive knowledge* of the abortion law. We examined binary measures of knowledge of each specific legal ground, knowledge of any legal ground, and whether the respondent had comprehensive knowledge of the law (i.e., correctly identified that abortion is legal under the 4 circumstances indicated).^1^ Women who reported that abortion is not legal under any circumstances are not included as having comprehensive knowledge. Finally, a binary measure of knowledge of *facility-based abortion service availability* was assessed by asking women, “Do you know where a woman can access facility-based abortion services in the community where you live?” (Yes/No). These questions were asked to all women who completed the survey, regardless of age, marital status, or reproductive history.

Our primary independent variables were region, comprising Ethiopia's 11 regional and administrative states: Addis Ababa, Afar, Amhara, Benishangul-Gumuz (BG), Dire Dawa, Gambella, Harare, Oromiya, Southern Nations, Nationalities, and People's Region (SNNP),^2^ Somali, and Tigray, and urban and/or rural residence.

### 2.3 Data analysis

We first explored sample characteristics and calculated prevalence estimates by region and urban and/or rural residence, assessing differences in women's awareness and knowledge of abortion legality and facility-based abortion availability. We then examined bivariate distributions of regional and urban/rural residence by women's general awareness of the abortion law, knowledge of specific legal grounds, comprehensive knowledge of the abortion law and knowledge of facility-based service availability; statistical differences between regions and residence were assessed via one-way ANOVA tests and design-based F statistics, respectively. Finally, we examined the relationship between knowledge of legality and availability by assessing knowledge of facility-based abortion service availability among women who correctly reported at least 1 legal ground for abortion, and vice versa. We used design-based analyses to account for the complex sampling design, including accounting for clustering of women within EAs and applying survey weights to account for differential probability of selection, in order to generate nationally and regionally representative estimates. We did not further account for clustering of responses within households as all women in the household share the same probability of selection. As there was slightly fewer than 1 woman (0.98) per household among all sampled households, there was not significant evidence of clustering within households*.* We analyzed data using Stata 16.1 (Statcorp LP, College Station, TX).

## 3 Results

### 3.1 Sample characteristics

Among the 8837 women in our analytic sample, the mean age of respondents was 26.5 years, and the majority (65.8%) were married or cohabitating (Table 1). One-quarter completed secondary education or higher and 37.7% never attended school. Most respondents had children, and 24.8% had 5 or more. The majority of respondents identified as Orthodox (46.8%) or Protestant (23.5%), and 28.3% identified as Muslim. Most respondents resided in Oromiya (37.8%), Amhara (23.5%), and SNNP (19.2%), and approximately 2-thirds (67.3%) resided in rural areas.

**Table 1:** . Background characteristics among analytic sample of women aged 15 to 49 in Ethiopia, 2019 (% weighted, *N* unweighted)

		%	N
Total			8837
**Region**			
	Addis Ababa	6.1	847
	Afar	1.0	415
	Amhara	23.5	1560
	Benishangul-Gumuz	1.1	284
	Dire Dawa	0.5	361
	Gambella	0.3	347
	Harare	0.4	331
	Oromiya	37.8	1724
	SNNP	19.2	1612
	Somali	3.5	193
	Tigray	6.4	1163
**Residence**			
	Rural	67.3	5048
	Urban	32.7	3789
**Age (mean)**		26.5	
**Education**			
	Never attended	37.7	3047
	Primary	36.7	3094
	Secondary or higher	25.6	2692
**Marital status**			
	Married/ in union	65.8	5615
	Divorced/widowed	9.1	885
	Never married	25.1	2337
**Religion of household**			
	Protestant	23.5	1856
	Orthodox	46.8	4341
	Muslim	28.3	2455
	Other	1.4	141
**Parity**			
	0	32.6	3039
	1–2	26.1	2446
	3–4	16.5	1464
	5+	24.8	1882
**Abortion knowledge**			
	Aware of abortion law	26.6	2503
	Knowledge of any legal ground	22.1	2205
	Comprehensive knowledge of law	5.0	441
	Knowledge of facility-based service availability	30.0	2862

Nearly one-third of women nationally reported that they knew where to access facility-based abortion services in their community. Nationally, about one-quarter of women had general awareness of the abortion law, yet just 5 percent of women had comprehensive knowledge. (Table 2).

**Table 2:** . National and regional Levels of awareness and knowledge of the abortion law among women aged 15 to 49 in Ethiopia, 2019 (% weighted, *N* unweighted)

		All women				Among women who are aware of a law,% who know each condition of the law					All women	
			Awareness of any law			Rape	Risk to the woman	Fetal impairment	Maternal disability	No circumstances*	Comprehensive knowledge **	
		N	%	*p*-value	N	%	%	%	%	%	%	*p*-value
**National**		8837	26.6		2503	55.9	56.6	43.5	36.5	6.6	5.0	
**Urban/rural**				**<0.0001**								**<0.0001**
	Urban	3789	34.8		1402	65.6	64.0	54.9	51.5	2.2	9.9	
	Rural	5048	22.6		1101	48.6	51.0	35.0	25.3	9.9	2.6	
**Region**				**<0.0001**								**<0.0001**
	Addis	847	45.4		383	64.0	68.6	53.7	45.4	2.1	9.7	
	Afar	415	11.8		51	70.0	53.6	16.7	19.4	0.0	0.3	
	Amhara	1560	23.1		369	67.1	57.3	50.9	41.6	1.5	4.7	
	BG	284	17.0		45	76.9	64.6	73.8	60.0	0.0	8.3	
	Dire Dawa	361	37.6		136	68.2	81.0	58.7	57.8	3.2	17.0	
	Gambella	347	17.9		59	80.4	52.6	40.7	46.3	5.3	5.9	
	Harare	331	36.6		116	98.2	96.7	91.3	75.5	0.0	26.6	
	Oromiya	1724	29.8		538	38.7	54.8	37.0	29.0	10.1	4.5	
	SNNP	1612	20.8		352	63.0	53.6	46.4	39.4	11.0	4.4	
	Somali	193	2.4		5	–	–	–	–	–	0.7	
	Tigray	1163	36.7		449	82.7	50.0	36.4	39.7	1.5	6.4	

*No circumstances: respondent does not think abortion is legal under any circumstance.

**Comprehensive knowledge: respondent correctly identifies all conditions of the abortion law.

*p*-values based on one-way ANOVA test (region) and design-based F statistic (urban/rural).

### 3.2 Regional variation of general awareness of abortion legality and availability

**Fig. 1:**
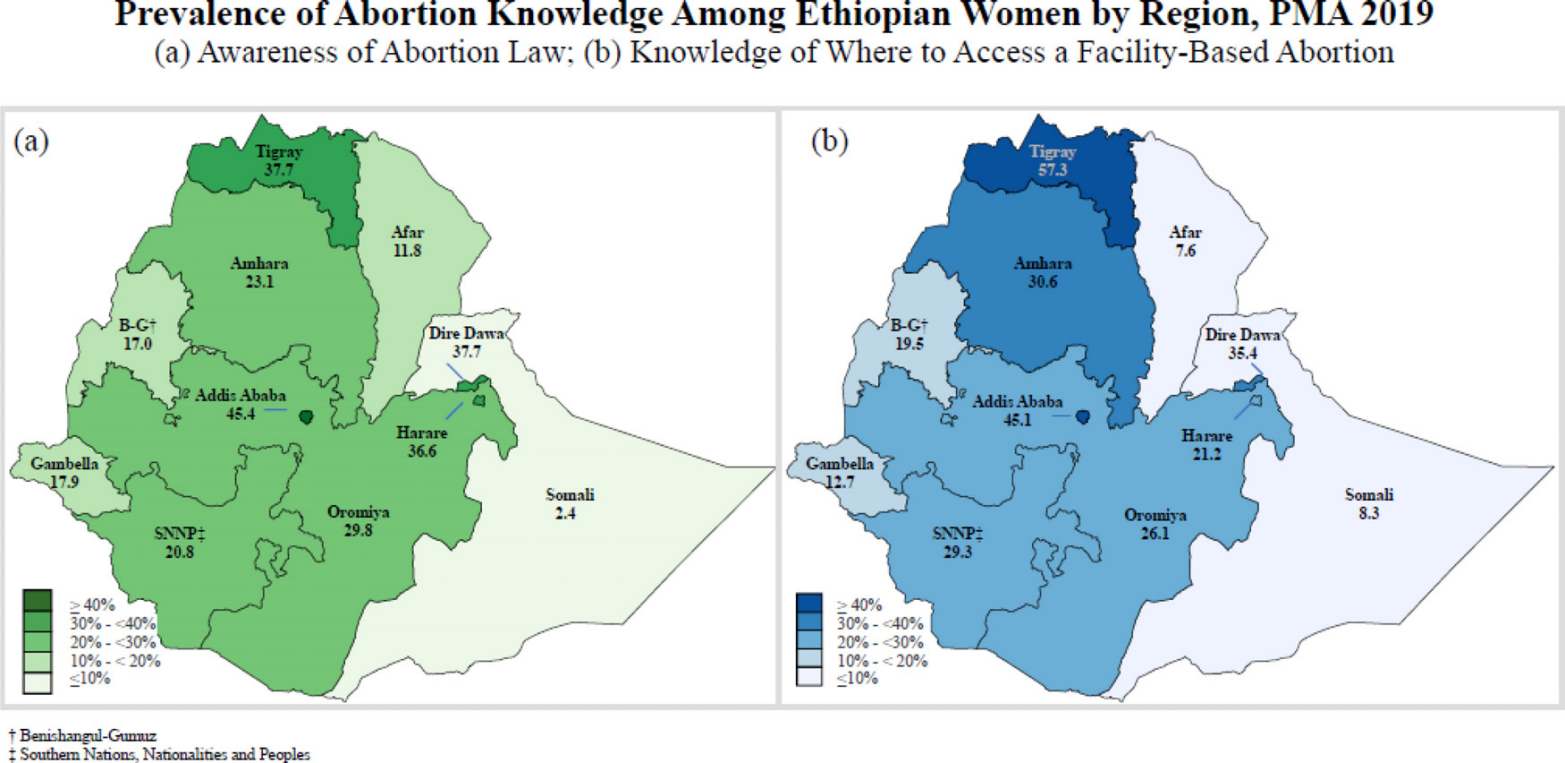
Knowledge of legality and facility-based abortion service availability by region among women aged 15 to 49 in Ethiopia, 2019.

Figure 1 displays the geographic distribution of *general awareness* of abortion legality, and *knowledge of facility-based abortion service availability*. Awareness of legality ranged widely from 45.4% in Addis Ababa to 2.4% in Somali. Knowledge of facility-based abortion service availability followed similar regional trends, with the highest prevalence in Addis Ababa (45.1%) and the lowest in Afar (7.6%) and Somali (8.3%). In most regions, more women knew where to access a facility-based abortion than were aware of an abortion law, with the exception of Dire Dawa, Harare, and Oromiya. Awareness of legality and knowledge of facility-based abortion service availability follow similar patterns across the country, with greater awareness and knowledge in urban areas and central and northern areas of the country.

Knowledge of where to access facility-based abortion services also varied considerably by region. The highest level of knowledge was in Tigray, where 57.3% of women knew where to access facility-based abortion services, and similar levels of knowledge were observed in Addis Ababa (45.1%). The lowest levels of knowledge were concentrated Afar (7.6%) and Somali (8.3%).

**Fig. 2:**
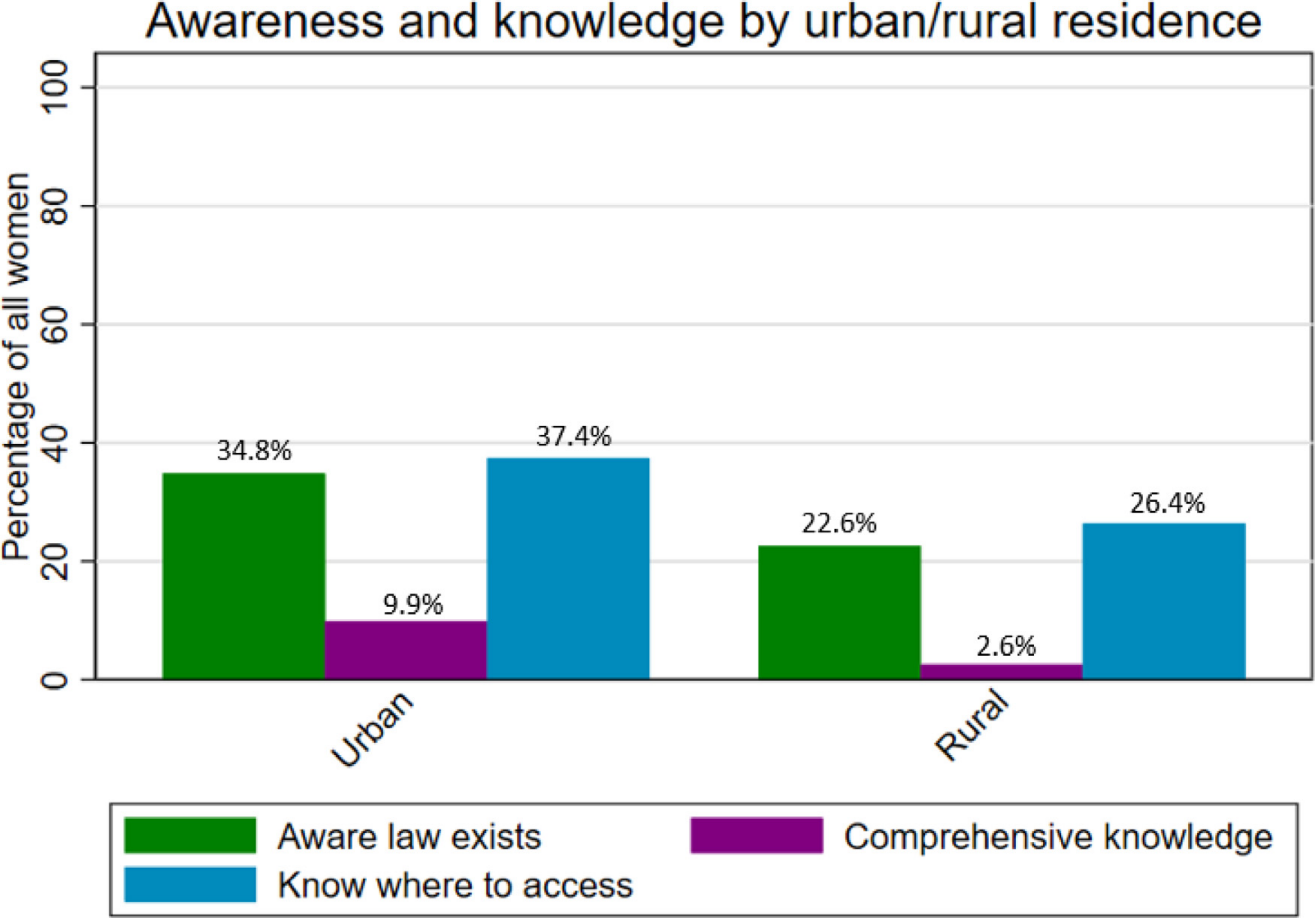
Awareness and knowledge of abortion legality and knowledge of facility-based abortion service availability by residence among women aged 15 to 49 in Ethiopia, 2019.

Figure 2 displays general awareness and comprehensive knowledge of legality, and knowledge of facility-based abortion service availability by urban/rural residence. Awareness and knowledge were consistently lower in rural, compared to urban, settings. More women knew about facility-based abortion care than were aware or knowledgeable about the law, regardless of residence.

### 3.3 Regional variation in comprehensive knowledge

Among women who knew there was a national law on abortion, more than half were aware that abortion is legal in cases of rape (55.9%) and when the woman's life is at risk (56.6%) (Table 2). Less than half were aware that abortion is legal in cases of fetal impairment (43.5%) or maternal disability (36.5%). Only 6.6% of women who knew a law existed reported that abortion was not legal under any circumstance.

Regional variation in knowledge of specific circumstances of the abortion law was considerable; knowledge of legality in cases of rape was highest (known by 55.9% of women who knew an abortion law exists) in all regions, except Oromiya (38.7%). Knowledge of legality in cases where a woman's life is at risk also varied by region, but was relatively high, ranging from 52.6% in Gambella to 96.7% in Harare. There was greater variation for legality in cases of fetal impairment or maternal disability, although knowledge of both was lowest in Afar and highest in Harare. Knowledge of fetal impairment ranged from 16.7% in Afar to 91.3% in Harare, and maternal disability ranged from 19.4% in Afar to 75.5% in Harare. As only 5 women in Somali reported general awareness of the law, we did not assess the percentage of women who were able to identify specific components of the law.

Comprehensive knowledge of the law also varied considerably by region and did not follow a pattern consistent with awareness. In urban areas, 9.9% of all women had comprehensive knowledge of the law, compared to just 2.6% of women in rural settings. Comprehensive knowledge was highest in parts of the country with large urban populations and high literacy rates, including Harare (26.6%) and Dire Dawa (17.0%), although was slightly lower (9.7%) in Addis Ababa, the country's largest urban center. Comprehensive knowledge was lowest in the remaining regions of the country, with fewer than 5 percent of women having comprehensive knowledge in Afar, Amhara, Oromiya, SNNP, and Somali.

### 3.4 Association between knowledge of abortion legality and availability

Table 3 presents national and sub-national estimates of the relationship between correct knowledge of any legal ground for abortion and knowledge of facility-based abortion service availability. Among women who correctly reported any one of the legal grounds for abortion, 63.3% also knew where to access care. In contrast, among women who knew where to access care, fewer than half (46.5%) correctly reported any legal ground for abortion. This relationship varied significantly by regional and urban/rural residence. In most regions, more women who knew the law also knew where to access care; however, this relationship was reversed in Afar, Gambella, and Harare.

**Table 3:** . Regional variation in knowledge of the law relative to knowledge of facility-based abortion service availability among women aged 15 to 49 in Ethiopia, 2019

			Among women who know any legal ground for abortion,% who know where to access an abortion			Among women who know a place to access an abortion,% who know any legal grounds for abortion	
		N	%	*p*-value	N	%	P-value
**National**		2204	63.27		2862	46.54	
**Urban/Rural**				**0.01**			**<0.001**
	Urban	1300	65.20		1588	55.67	
	Rural	905	61.53		1274	40.36	
**Region**				**<0.001**			**<0.001**
	Addis	360	63.25		379	59.89	
	Afar	50	55.86		31	85.24	
	Amhara	347	65.30		483	46.65	
	BG	37	64.11		52	47.43	
	Dire Dawa	124	64.33		128	61.35	
	Gambella	53	45.06		46	58.51	
	Harare	115	43.24		80	74.35	
	Oromiya	390	50.83		447	41.00	
	SNNP	302	74.38		482	45.65	
	Somali	5	–		20	20.04	
	Tigray	421	88.11		714	52.23	

*p*-value based on one-way ANOVA test (region) and design-based F statistic (urban/rural).

## 4 Discussion

Our study aimed to quantify national, regional, and residential awareness of abortion legality, comprehensive knowledge of the abortion law, and knowledge of facility-based abortion service availability. Findings indicate that knowledge of abortion legality and availability is low, with considerable regional and residential heterogeneity.

Women's limited knowledge of the circumstances under which abortion is legal warrants further attention. Reproductive health stakeholders in Ethiopia have described confusion among women and providers alike about the conditions under which abortion is legal; this uncertainty impedes use and provision of safe abortion services [20]. In Ethiopia, abortion legality reform was pursued quietly to avoid stoking anti-abortion sentiment, however this “strategy of silence” has contributed to gaps in knowledge surrounding abortion availability and legality [20]. Promoting information about abortion legality is challenging, and at times may be less salient to access than women's knowledge that safe abortion services are available, especially in a large, geographically diverse setting like Ethiopia. Advocates must often balance the benefits of publicizing legal reforms to increase public awareness, with striving to minimize anti-abortion backlash.

The heterogeneity we observe by region and residence aligns with patterns observed for other reproductive health indicators, including abortion rates [7, [21], [22], [23]]. We find higher awareness and knowledge among women living in smaller administrative regions (including Addis Ababa, Harare, and Dire Dawa), which have better access to comprehensive abortion care and are predominantly urban. These regions also have higher abortion rates than most other parts of the country, according to facility-based estimates, which may, in part, reflect travel by women in rural areas to facilities in urban areas for care [7, 12]. According to Ethiopia's 2016 Demographic and Health Survey, the prevalence of abortion ranges across the country, with 11.3% of women in Tigray reporting ever having had an abortion, compared to 4.5% in Benishangul-Gumuz, and higher self-reported prevalence among women in rural (9.2%) than urban (6.7%) areas [21]. Despite higher abortion prevalence in rural areas [21], we find lower levels of abortion-related knowledge in rural settings, suggesting a need for greater dissemination of information regarding legality and availability in rural areas. Residential variation in knowledge of the law echoes results from other countries, where women living in rural areas had lower awareness and knowledge of national abortion laws [2].

Considerable regional and residential disparities in abortion-related knowledge continue to persist in Ethiopia, highlighting important implications for services in the country. The government of Ethiopia committed to improving health equity as a major goal in their 2015 Health Sector Transformation Plans [24, 25], and the regional disparities revealed in our study offer important benchmarks for health officials and policymakers to understand the current status of inequities in abortion-related knowledge, which can guide future programs and policies.

We also find discrepancies between knowledge of legal grounds and knowledge of facility-based abortion service availability; more women know *where* to access an abortion than under what legal circumstances they can do so. In some settings, particularly those with lower literacy and/or educational attainment, women's knowledge of abortion availability may be more salient to their ability to access to safe abortion care, if needed, than legal knowledge. Efforts to improve women's knowledge of abortion legality and availability are needed to enhance access to safe abortion and reduce unsafe abortion complications. In particular, bolstering women's comprehensive knowledge of the abortion law could facilitate women's ability to advocate for their legal right to safe abortion care. Interventions should be regionally-tailored and target specific geographies (e.g., Afar and Somali) to address inequities in knowledge. While not explored in our study, future research should examine regionally specific issues pertaining to the affordability and accessibility of safe abortion care, both within and outside facility settings, which may also shape women's access to care.

### 4.1 Limitations

We asked respondents about their knowledge of facility-based abortion services only; thus, we are unable to capture knowledge of availability outside health facilities. In the corresponding survey question, “facility” and “community” were open to interpretation by the respondent, and could refer to a range of service delivery points and community groupings. Additionally, our questionnaire did not ask respondents whether abortion is legal for women younger than 18, which is a component of the Ethiopian abortion law, thus we are not able to capture knowledge in this regard [8].

We find significant regional disparities in knowledge of abortion legality and availability in Ethiopia. Knowledge of these concepts is imperative for ensuring access to safe abortion care, especially in contexts like Ethiopia where abortion is legal in a limited range of circumstances. Efforts to improve women's knowledge of legality and availability of safe abortion care should be tailored to the local context. Our findings can guide programs and policies dedicated to expanding safe abortion access in Ethiopia.

## Footnotes

1 Of note, the 2005 legal amendment in Ethiopia also makes abortion legally available for women under age 18, but due to an error in questionnaire design, we did not collect information on this aspect of the law.

2 In 2020, a new region (Sidama) was formed from the SNNP region. This was done after the initial design and implementation of our study and thus we refer to the original 11 regions and states that existed at the time of implementation.
